# The Grey Mouse Lemur Uses Season-Dependent Fat or Protein Sparing Strategies to Face Chronic Food Restriction

**DOI:** 10.1371/journal.pone.0008823

**Published:** 2010-01-21

**Authors:** Sylvain Giroud, Martine Perret, Peter Stein, Joëlle Goudable, Fabienne Aujard, Caroline Gilbert, Jean Patrice Robin, Yvon Le Maho, Alexandre Zahariev, Stéphane Blanc, Iman Momken

**Affiliations:** 1 Unité Mixte de Recherche 7178, Département d'Ecologie Physiologie Ethologie, Institut Pluridisciplinaire Hubert Curien, Centre National de la Recherche Scientifique, Université de Strasbourg, Strasbourg, France; 2 Mécanismes Adaptatifs et Evolution, Unité Mixte de Recherche 7179, Centre National de la Recherche Scientifique, Muséum National d'Histoire Naturelle, Brunoy, France; 3 Department of Surgery, University of Medicine and Dentistry of New Jersey, Stratford, New Jersey, United States of America; 4 Institut des Sciences Pharmaceutiques et Biologiques, Faculté de Pharmacie, Lyon 1, Fédération de Biochimie, Hôpital Edouard Herriot, Lyon, France; 5 Université Henri Poincaré, Nancy Université, Vandoeuvre-Les-Nancy, France; Pennsylvania State University, United States of America

## Abstract

During moderate calorie restriction (CR) the heterotherm *Microcebus murinus* is able to maintain a stable energy balance whatever the season, even if only wintering animals enter into torpor. To understand its energy saving strategies to respond to food shortages, we assessed protein and energy metabolisms associated with wintering torpor expression or summering torpor avoidance. We investigated body composition, whole body protein turnover, and daily energy expenditure (DEE), during a graded (40 and 80%) 35-day CR in short-days (winter; SD40 and SD80, respectively) and long-days (summer; LD40 and LD80, respectively) acclimated animals. LD40 animals showed no change in fat mass (FM) but a 12% fat free mass (FFM) reduction. Protein balance being positive after CR, the FFM loss was early and rapid. The 25% DEE reduction, in LD40 group was mainly explained by FFM changes. LD80 animals showed a steady body mass loss and were excluded from the CR trial at day 22, reaching a survival-threatened body mass. No data were available for this group. SD40 animals significantly decreased their FM level by 21%, but maintained FFM. Protein sparing was achieved through a 35 and 39% decrease in protein synthesis and catabolism (protein turnover), respectively, overall maintaining nitrogen balance. The 21% reduction in energy requirement was explained by the 30% nitrogen flux drop but also by torpor as DEE FFM-adjusted remained 13% lower compared to *ad-libitum*. SD80 animals were unable to maintain energy and nitrogen balances, losing both FM and FFM. Thus summering mouse lemurs equilibrate energy balance by a rapid loss of active metabolic mass without using torpor, whereas wintering animals spare protein and energy through increased torpor expression. Both strategies have direct fitness implication: 1) to maintain activities at a lower body size during the mating season and 2) to preserve an optimal wintering muscle mass and function.

## Introduction

Torpor is associated with a profound reduction of energy expenditure to as little as 3% of the euthermic rates at ambient temperature [1]. The fitness advantages of torpor are two-fold. It likely improves survival during periods of food shortage or reproductive rest [2], [3] and may increase reproductive success following these periods [4], [5].

Energy and water conservation through torpor is likely to be essential for surviving prolonged periods of restricted energy availability. Nevertheless more energy conservation may not necessarily be more advantageous. High rates of metabolism were hypothesized to be beneficial for endotherms when resources are abundant [6].

The torpor avoidance may be associated to the several reported negative physiological consequences of torpor arousal, such as a possible increase in oxidative stress as we [7] and others [8] recently observed. The accumulating evidence for important and widespread costs of torpor raises the question of whether the assumption that optimal energy economy necessarily involves maximizing the depth and duration of torpor bouts is valid. An alternative strategy might be linked to the type of energy stores used as fuel during the period of food shortage. In particular, a reduction in the metabolically active fat-free mass *vs.* metabolically inactive fat mass would represent a significant strategy to lower energy requirements without cessation of activities.

Seasonal heterotherms accumulate and subsequently loose energy stores in a yearly body mass gain-loss cycle [9]–[12] and most of the increase in mass is due to an increase in fat reserves. During periods of food scarcity, endogenous lipids are the main source of energy utilized and proteins are spared [13], [14], through an increase in lipolysis and ketogenesis [15], [16]. Hibernators spontaneously experience a starvation-like state during the phase of body mass loss of their weight cycle [13] and spare protein during this period, as reported in hibernating ground squirrels under a very low calorie diet [14]. Conversely, energy-deprived hibernators in summer may experience “fat sparing”, as supported by studies in food-deprived Belding and arctic ground squirrels [17]. These studies on protein or fat sparing were mainly conducted on rodent species, but to date, no data are available on primates.

**Figure 1:**
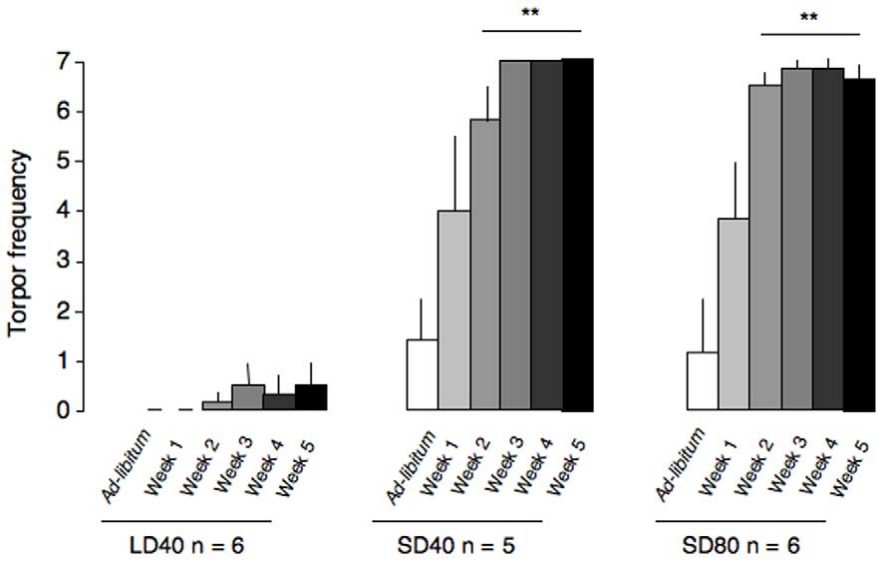
Changes in Torpor frequency in food-deprived mouse lemurs. Torpor frequency changes during a 5-week food restriction in long-days and short-days mouse lemurs exposed to a 40% food deprivation (LD40 and SD40, respectively) and in short-days 80% calorie restricted animals (SD80) (From Giroud et al. 2009 [61]). Body temperature was recorded by using a small data logger (model TA10TA-F20, 3.2 g; DSI, St Paul, MN), which was implanted in the abdominal cavity of each animal. Torpor was defined when body temperature of mouse lemurs dropped below 33°C. Torpor frequency (0 to 7) represents the number of occurrence of torpor bouts during a week. By using a generalized linear model, we tested the differential time course of torpor frequency over the 5 weeks of food deprivation between the 3 groups of mouse lemurs. In each group, Bonferroni tests compared weeks of food restriction with the control (*ad-libitum*) value. Values are means ± SE. **p<0.01.

The grey mouse lemur (*Microcebus murinus*) is among the rare known heterothermic primates and shows marked seasonal changes in body mass in response to the Malagasy predictable cycle of food allocations. During the summer season, mouse lemurs are in an active reproductive state. After autumnal fattening, animals increase their torpor propensity for energy and water conservation, to cope with the drastic food shortages during the dry and cold season. We investigated the pattern of torpor expression of this small Malagasy prosimian, as a function of food availability [18]. We reported that only wintering mouse lemurs increase their torpor propensity, either during moderate (40%) or severe (80%) calorie restriction [18] (Figure 1). Conversely, animals in the summer phenotype, when energy intake is moderately restricted by 40%, show little change in their torpor patterns and are able to stabilize their body mass at a lower level. Similar the hibernators, the grey mouse lemur would share some common mechanisms involved in the type of energy stores mobilized during food restriction. Given that the energy costs of protein turnover can account for 20 to 40% of the basal metabolic rate [19], any modulation of the fat-free mass may represent an important strategy of energy economy that will maintain fitness at a lower body size.

Knowing the changes in the rate of whole body protein turnover, energy expenditure, and body composition that accompany torpor expression during winter and non-use of torpor during summer is essential to further understand the nature and the limits of the strategies of energy economy used by the grey mouse lemur to face predicted and unpredicted food shortages and thus regulate energy balance. The present study extended our previous observations by investigating the protein-energy interrelationship during a graded calorie restriction in the winter and summer acclimated mouse lemurs [18]. We specifically hypothesized that this species would use either protein-sparing or fat-sparing strategies to adjust energy expenditure to the behavioral requirements of a given season.

## Materials and Methods

### Ethics Statement

The Research was conducted under the authorization 67-223 from the Direction Départementale des Services Vétérinaires du Bas-Rhin and the Internal Review Board of the UMR 7179.

### Animals

The 34 adult male grey mouse lemurs (*Microcebus murinus*, Cheirogaleidae, Primates) used in this study were born in the laboratory breeding-colony of Brunoy (UMR7179 CNRS/MNHN, France; European Institutions Agreement # 962773) from a stock originally caught along the southwestern coast of Madagascar 40 years ago. Seasonal Malagasy rhythms were reproduced by alternating 6-month periods of long-days (light∶dark 14∶10) and short-days (light∶dark 10∶14). In *M. murinus*, an exposure to long-days triggers a summer-like state, i.e. stimulates reproductive and locomotor's activities, increases metabolic rates and reduces body mass. Conversely, an exposure to short-days sets-up a winter-like phenotype in mouse lemurs, by triggering a fattening process, by increasing food intake, and by reducing metabolic rates and locomotor's activities [10], [20], [21]. Mouse lemurs were transferred in our laboratory at Strasbourg (UMR7178 CNRS/UdS, France) and housed individually in cages (70×68×52 cm), visually separated from each other, in order to minimize social influences. The relative humidity in animal rooms was maintained constant (55%), mouse lemurs were kept at ambient room temperatures of 25°C, and were under to the same photoperiodic regimen that they were exposed at Brunoy, i.e. long-days (LD) and short-days (SD) exposures, respectively.

### Energy Intake and Calorie Restriction

After a month of acclimatization to their new environment, individual calorie intakes were measured during a 10-day period, in order to calculate subsequent food-restricted energy allotments. Animals were fed, in *ad-libitum* conditions, on fresh banana and a standardized homemade mixture containing baby cereals, spice bread, egg, concentrated milk, white cheese, vitamins and dietary minerals (Vitapaulia/MR, Intervet, France and Toison d'or, Clément Thékan, France). Since grey mouse lemurs, and particularly those under winter phenotype, tend to overfeed when isolated and thus gain mass during the *ad-libitum* period, energy intake was clamped to the level required to stabilize their body mass. This was necessary to avoid significant underestimation of the calorie restriction (CR) needed for the test diets. Each individual was initially fed *ad-libitum* with banana and the homemade mixture and progressively, daily energy intake was narrowed according to the body mass time-course, as already done in our previous study [18].

Half of the animals in each photoperiod were then provided with 60% ( = 40% CR) or 20% ( = 80% CR) of these individually derived energy requirements, during 35 days. Food-restricted allotments were available every day at the onset of the dark phase. Water was always provided *ad-libitum*. Daily food intake was calculated from the difference between provided and remaining food weights and was corrected for dehydration. Energy equivalents of 3.7 kJ/g for the banana and 4.6 kJ/g for the mixture were used to convert grams of food intake to kJ. During the 35-day food restriction period, mouse lemurs in the 40% CR received an energy allotment of either 47.5±1.3 kJ/day (LD40, long day, 40% restriction) or 45.8±3.3 kJ/day (SD40, short day, 40% restriction). The 80% food-restricted LD (LD80) and SD animals (SD80) were provided with an energy allocation of 16.5±2.5 kJ/day and 15.5±0.6 kJ/day, respectively. The LD80 mouse lemur group weighed 78±2 g under *ad-libitum* diet weighed, 52±1 g after 22 days on the 80% restriction diet. According to the data from the Brunoy colony, weights of 50 g are survival threatening for this photoperiod (21). Therefore, these animals were excluded from the study before the end of the food-deprived trial, replaced in the breeding colony and re-fed with an *ad-libitum* diet. No urine samples were collected or energy measurements were performed in the CR period for this group.

### Protocol Overview

Each animal was studied during the *ad-libitum* period and again after 35 days of CR. The tests were identical in both conditions and consisted of the measurement of daily energy expenditure (DEE), fat-free mass (FFM), fat mass (FM) and water flux rate (rH_2_0) by the doubly labeled water (DLW) method, resting metabolic rate (RMR) by respirometry, protein turnover by using [^15^N]-glycine and catecholamine concentrations in a 24-hr pooled urine. Body temperature was continuously recorded by telemetry during the *ad-libitum* and food-deprived period.

### Daily Energy Expenditure, Body Composition and Water Turnover

DEE was determined during a 2-day period by the multipoint DLW methodology [22]. A baseline urine sample was quickly collected from gentle pressure on the bladder and a premixed 2 g/(kg estimated total body water, TBW) dose of DLW was intravenously injected to the animals. The dose was composed of 0.55 g/(kg estimated TBW) 97% H_2_^18^O (Rotem Industries Ltd., Israel) and 0.15 g/(kg estimated TBW) 99.9% ^2^H_2_O (Cambridge Isotope Laboratories, Andover, MA, USA) and was diluted with 3% NaCl to physiological osmolarity. We assumed a percentage of hydration of 0.60 and 0.55 for LD and SD animals, respectively, to calculate doses. The doses were calculated to ensure an *in vivo* enrichment of about 250 and 1200‰ for 18-oxygen and deuterium, respectively [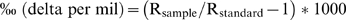 with R being the ratio heavy to light isotope]. Isotopic equilibration in body water was determined from a blood sample collected at 1-h post-dose from quick sampling of the saphenous vein. Immediately after collection, blood-containing capillaries were rapidly flame-sealed. The mouse lemur was then released inside its own cage and urine samples were collected in cryogenically stable tubes 24 and 48 h after blood collection. Blood and urine samples were respectively stored at 5°C and −20°C until analyses by isotope ratio mass spectrometry.

Water from serum and urine samples were extracted by cryo-distilation, as previously described [23]. 0.1µL of water was reduced to hydrogen and carbon monoxide by reduction on a glassy carbon reactor held at 1400°C in an elemental analyzer (Flash HT; ThermoFisher Germany). Hydrogen and carbon monoxide gases were separated by a GC column held at 104°C coupled to a continuous-flow Delta-V isotope ratio mass spectrometer. Isotopic abundances of deuterium and 18-oxygen in hydrogen and carbon monoxide gazes were measured in quintuplicate and repeated if SD exceeded 2 and 0.5‰, respectively. All enrichments were expressed against International Atomic Energy Agency standards.

CO_2_ production was calculated according to the single pool equation of Speakman [24]: 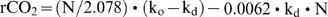, where N represents the average isotope dilution space of oxygen-18 calculated from Coward [25] by the plateau method using the 1-hour post-dose sample. k_o_ and k_d_ represents the isotope constant elimination rates calculated by linear regression of the natural logarithm of isotope enrichment as a function of elapsed time from day 1 samples. DEE was calculated by the Weir's equation [26] using a food quotient of 0.823 estimated from the animal's diet. Total body water (TBW) was measured from the dilution space of 18-oxygen after correction for exchange by the factor 1.007 [27]. FFM was calculated from TBW by assuming hydration coefficient of 73.2% that was shown not to be affected by chronic CR [28]. FM was calculated by the difference of FFM from the body mass. rH_2_0 was assessed by the multiplication of the average isotope dilution space of oxygen-18 (N) with the deuterium constant elimination rate (K_d_) and corrected for isotope fractionation [24].

### Resting Metabolic Rate (RMR)

Oxygen (O_2_) consumption was measured using an open-circuit respirometry system (Sable Systems International, Las Vegas, USA). The concentration of O_2_ in the outgoing air was successively measured in four cages (27×27×27 cm) including one cage left vacant as reference for the ambient gas concentrations. Measurements were performed continuously over 48 h, excluding a daily 20-min period required for calibration of O_2_ analyzer. Calculations of O_2_ consumption were derived from the second day of respirometry measurement, the first day being considered for mouse lemurs as a habituation period to confinement. The system was rinsed for 90 s between each measurement. Each cage was sampled during 180 s (1 sample per second) every 12 min and final values of O_2_ concentration was the mean of values recording during 60 s. Energy expenditure was calculated by using an energy equivalent of 20.1 J/ml O_2_. As mouse lemur shows marked daily rhythms of body temperature (Tb) and metabolic rate with an active state restricted during the dark phase. RMR was estimated during the resting normothermic period, which follows torpor bout and precedes the dark phase, and expressed as kJ.min^−1^. During all the measurement period, mouse lemurs were under an *ad-libitum* regimen, before the food-deprived trial, and partially (40 or 80%) food-deprived during the calorie-restriction.

### Nitrogen Balance and Protein Turnover

Animals were individually placed in metabolic cages for 24 hours after one week on the *ad-libitum* control diet and after the 35 days of calorie restriction. During those 24-hour periods, food intake was measured and cumulated feces and urine were collected on ice. Total nitrogen in urine was measured by chemo luminescence (Antek 7000, ALYTECH – Juvisy Sur Orge – France) and by the Kjedkhal method in feces and food, as previously described [29]. Nitrogen balance was calculated as the difference between nitrogen intake and the excretions in urine and feces.

During those 24 hours, protein turnover was determined by means of the [^15^N]-glycine end-product technique [30]. After the collection of basal urine samples and right before the dark phase, the animals were gently force-fed 7 mg/kg body weight of a [^15^N]-glycine solution. ^15^N-urea and ^15^N-amonia enrichments were measured in the 24-hr urine pools, as previously described [31]. Protein turnover was calculated according to a single-pool model. Nitrogen flux (Q) was calculated as Q = d/e where d is the dose given and e is the cumulated excretion of ^15^N end-products in urine. Synthesis and catabolism rates were then calculated from the following equation Q = S + E = C + I, where S is the synthesis rate of protein, E is excretion of nitrogen (urine plus feces), C is the catabolism rate, and I is dietary nitrogen intake.

### Metabolite Assays

Concentrations of normetanephrine and metanephrine were determined by high performance liquid chromatography with electrochemical detection [32] on the 24-hour urine samples.

### Data Analysis and Statistics

Throughout the analysis, the sample size of analyzed data varied to a small extent due to limitations imposed by the 24hr urine volume collected or to the difficulty encountered in collecting spot urine or blood samples, especially after calorie restriction. The exact sample sizes for each variable are indicated on the figures. Except body mass and torpor frequencies, all data were normally distributed and parametric tests were used. During the *ad-libitum* period, differences between LD and SD groups were assessed using a Student *t* test. In each animal group, Student paired *t* test compared the *ad-libitum* and food-restricted levels for each parameter studied. To determine differences between food-restricted animal groups, an analysis of variance was used and Fisher's protected least significant difference (PLSD) tests were performed. A generalized linear (GLZ) model, with gamma error distribution and log-link function, was used to analyze effects of photoperiod and calorie restriction intensity (40 *vs.* 80%) on the time courses of body mass, along the 35 days of food deprivation. Then, each time course of body mass was analyzed with a Friedman's analysis of variance. DEE and RMR were adjusted for differences in the active metabolic mass by analysis of covariance using FFM as covariate. All reported values are means ± SE, and p<0.05 was considered significant. All statistic analyses were performed by JMP (V5.1.1, NC, USA), except for the GLZ model that was realized with Statistica (V7.1.515.0, Statsoft, France).

## Results

### Body Mass and Composition

**Figure 2:**
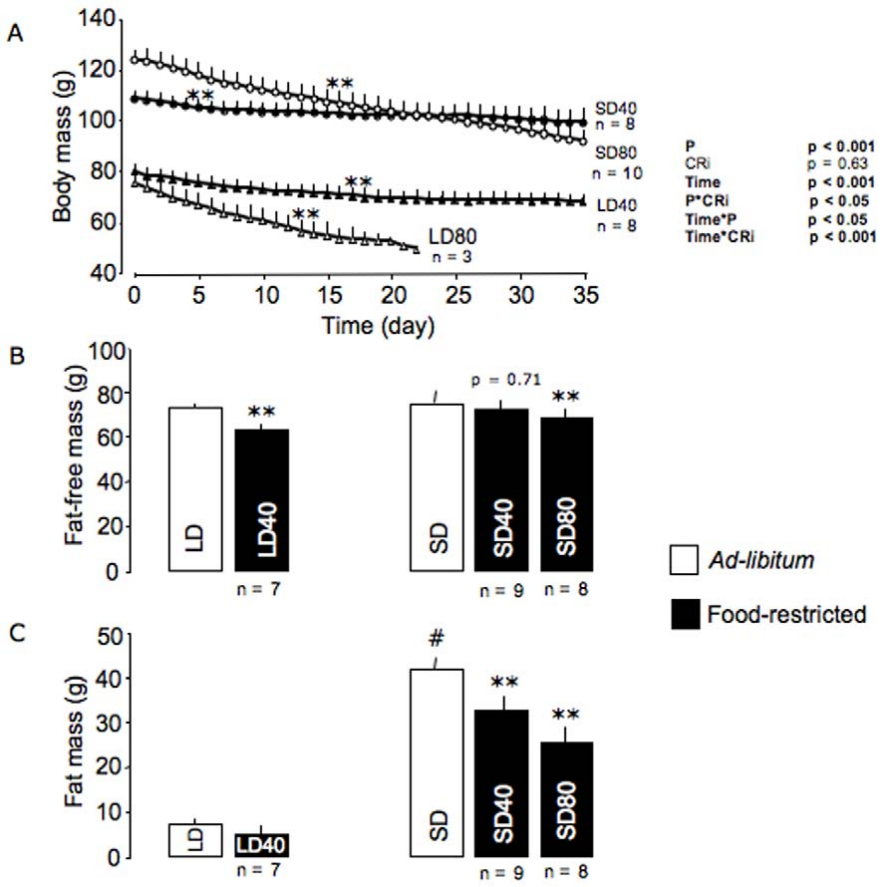
Modifications of body mass and composition in food-restricted mouse lemurs. Body mass time courses (A) and, changes in fat-free mass (B) and fat mass (C) during 5 weeks of food deprivation. The statistics, mentioned on the right side of the graph, show overall effects of photoperiod (P) and calorie restriction (CRi) on the body mass time courses during a 35-day food deprivation (time) in long-days (LD) and short-days (SD) mouse lemurs under 40% (LD40 and SD40, respectively) and 80% food restriction (LD80 and SD80, respectively). Please, note that LD80 animals were excluded at *day 22*, before the end of the food-restricted trial. Values are expressed as means ± SE. # LD *vs.* SD groups under *ad-libitum*. **p<0.01.

Under *ad-libitum* condition, LD and SD mouse lemurs displayed significant different body mass levels of 79±2 and 118±3 g, respectively (t = −7.47, p<0.001; Figure 2A). During food restriction, an overall decrease in body mass was found (F = 161.4, df = 22, n = 23, p<0.001). Mouse lemurs under 40% CR (LD40 and SD40) and those under 80% CR (LD80 and SD80) had significantly reduced body mass after food restriction (LD40: ℵ^2^ = 228.0, p<0.001; LD80: ℵ^2^ = 64.1, p<0.001; SD40: ℵ^2^ = 102.1, p<0.001; SD80: ℵ^2^ = 347.2, p<0.001) at a respective average rate of −0.3±0.1 and −0.9±0.1 g.day^−1^, values that significantly differed from each other (p<0.001).

During the *ad-libitum* period, FFM of LD and SD mouse lemurs did not differ from each other (73±2 *vs.* 76±1 g, t = −1.0, p = 0.34; Figure 2B). Conversely, FM was higher in SD than in LD animals (7±2 *vs.* 42±3 g, t = −11.5, p<0.001; Figure 2C). During the calorie restriction period, SD40 mouse lemurs did not reduce their FFM, but significantly decreased their FM level by 21%. As a result, FFM and FM levels represented 69 and 31% of body mass after CR, respectively. Conversely, SD80 and LD40 food-restricted animals displayed a significant 13% and 12% decrease in FFM levels, respectively, reaching values of 68±2 and 64±3 g. LD40 group had only a 31% decrease in FM that did not reach significance (p = 0.11), whereas SD80 mouse lemurs reduced by 47% FM.

### Water Turnover

**Figure 3:**
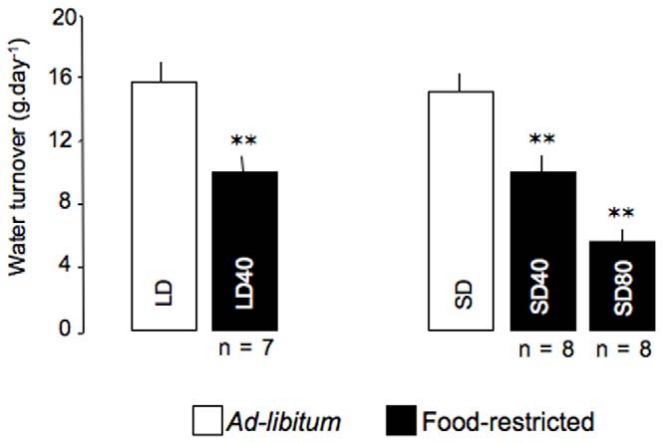
Water turnover changes induced by a 5-week food restriction in mouse lemurs. LD and SD: long-days and short-days mouse lemurs, respectively. LD40 and SD40: LD and SD animals under a moderate 40% calorie restriction, respectively. SD80: SD mouse lemurs facing a severe 80% food deprivation. Values are means ± SE. **p<0.01 *vs. ad-libitum* value.

Under *ad-libitum* condition, no differences in water turnover rate were found between LD and SD mouse lemurs (t = 0.5, p = 0.64, Figure 3). LD40 and SD40 food-restricted mouse lemurs significantly reduced their rH_2_O levels by 37% and 32%, respectively. There was a 2-fold larger (63%) decrease in rH_2_O (p<0.01) with the SD80 group.

### Daily Energy Expenditure and Resting Metabolic Rate

**Figure 4:**
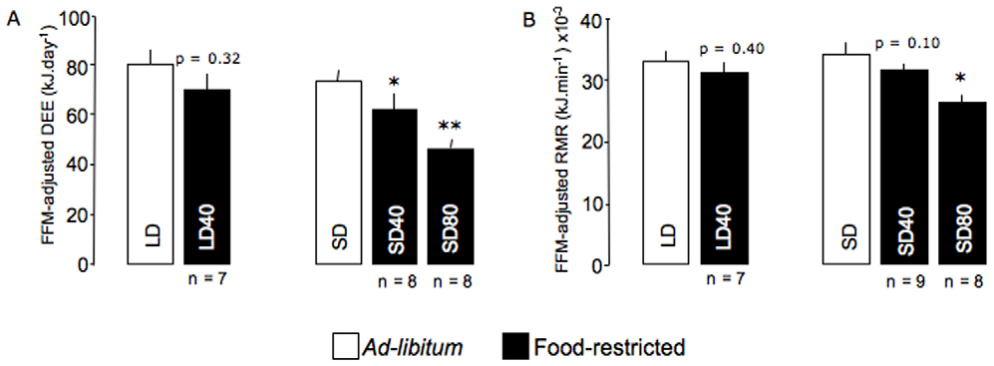
Food-restriction induced changes in energy expenditures in mouse lemurs. Changes in fat-free mass (FFM)-adjusted daily energy expenditure (DEE, A) and resting metabolic rate (RMR, B) in long-days (LD) and in short-days (SD) mouse lemurs under 40% food restriction (LD40 and SD40, respectively) and SD animals facing an 80% calorie restriction (SD80). Values are means ± SE. *p<0.05, **p<0.01 *vs. ad-libitum* value.

LD and SD mouse lemurs did not show any differences in FFM-adjusted DEE (DEE_FFM_), under an *ad-libitum* diet (80.1±6.2 *vs.* 75.8±4.2 kJ.day^−1^, p = 0.58, n_LD_ = 7, n_SD_ = 17). With food deprivation, all mouse lemurs decreased their DEE by 25, 21 and 47% in LD40, SD40 and SD80 groups, respectively. For the LD40, the reduction in DEE could be accounted for the reduction in FFM, as DEE_FFM_ after calorie restriction was no longer different from *ad-libitum* value (p = 0.32) (Figure 4A). Conversely DEE_FFM_ from SD40 and SD80 animals remained significantly lower after food deprivation by 13 and 40%, respectively.

During the *ad-libitum* period, FFM-adjusted RMR did not differ between photoperiods (0.033±0.02 *vs.* 0.034±0.01 kJ.min^−1^, p = 0.51, n_LD_ = 7, n_SD_ = 17). After adjustment for FFM, only the SD80 animals showed a significant (23%) reduction in RMR after calorie restriction (Figure 4B).

### Nitrogen Balance and Flux

**Figure 5:**
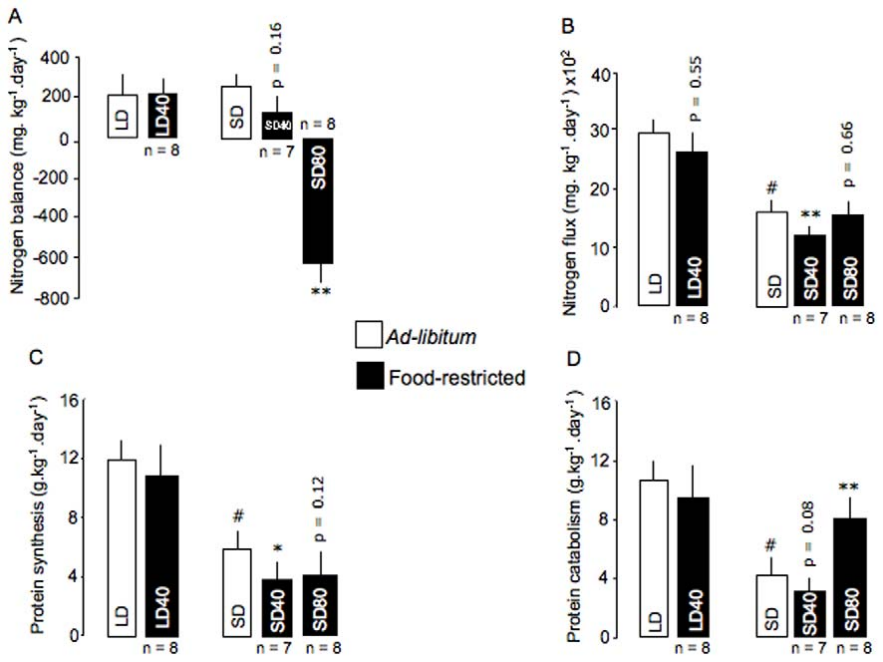
Changes in nitrogen balance and flux, and protein turnover in food-deprived mouse lemurs. Changes in mass-specific nitrogen balance (A), nitrogen flux (B), protein synthesis (C) and catabolism (D), normalized by body mass, in long-days (LD) mouse lemurs under a moderate 40% food deprivation (LD40) and in short-days (SD) animals under a 40% or an 80% calorie restriction (SD40 and SD80, respectively). Values are means ± SE. # LD *vs.* SD groups under *ad-libitum*. *p<0.05, **p<0.01 *vs. ad-libitum* value.

Under *ad-libitum* condition, mass-specific nitrogen balance was positive around 200 mg/kg/d and did not differ between LD and SD mouse lemurs (Figure 5A). After food restriction, no changes were further noted in the LD40 and SD40 animals. Only in SD80 animals mass-specific nitrogen balance decreased by 800 mg/kg/d became strongly negative (p<0.001). During the *ad-libitum* period, mass-specific nitrogen flux (Figure 5B) was significantly higher in LD than in SD mouse lemurs (2958±206 *vs.* 1615±145 mg.kg^−1^.day^−1^, t = 5.33, p<0.001). After food restriction, only SD40 animals significantly reduced their mass-specific nitrogen flux by 30% on average.

### Protein Turnover

Under *ad-libitum* condition, the rates of protein synthesis and breakdown significantly differed between photoperiods (Figures 5C and 5D, respectively). In LD40 mouse lemurs, both protein synthesis and breakdown remained unaffected after the 35 days of CR. After severe calorie restriction the loss in body protein observed in winter-acclimated mouse lemurs was essentially explained by a 130% increase in breakdown without significant changes in synthesis. Conversely, the rates of protein synthesis and catabolism were reduced in food-restricted SD40 mouse lemurs, by 35 and 39% respectively, although non-significant for protein catabolism (p = 0.08).

### Catecholamines

During the *ad-libitum* period, no difference was reported between LD and SD mouse lemurs in catecholamine levels **(** **Table 1** **)**. After food deprivation there was a threefold increase in normetanephrine and metanephrine in the SD80 animals.

**Table 1:** Catecholamine levels in long-days (LD) and short-days (SD) mouse lemurs under *ad-libitum* diet (LD-AL and SD-AL, respectively) and under a 40% calorie restriction (LD40 and SD40, respectively) or an 80% food deprivation (SD80).

	NMN (g/mmol.creatinine^−1^)	MN (g/mmol.creatinine^−1^)
**LD-AL**	30.1±7.0	20.2±4.0
**LD40**	38.2±8.3	31.4±6.3
**SD-AL**	37.2±4.8	22.6±2.4
**SD40**	40.5±8.6	32.3±8.9
**SD80**	88.2±16.1*	57.7±14.2*

*p<0.05 *vs. ad-libitum* condition.

## Discussion

### Mouse Lemurs Spare Protein during ‘Winter’ but Fat in ‘Summer’ under Moderate Food Shortage

Under a moderate food shortage, mouse lemurs in ‘winter’ spared lean body mass, unlike animals in ‘summer’ who showed a reduction in fat-free mass. These results suggest that animals in the winter phenotype rely on fatty acids for energy during food restriction. This might be due to 1) the 6-fold larger amount of fat mass in mouse lemurs in winter compared to those in summer and 2) the seasonal metabolic shift in oxidation in the type of substrates under winter-acclimated phenotype.

Prior to hibernation, much of the increase in body mass of seasonal mammals is due to fatty acid storage, and this endogenous lipid reserves constitutes the primary source of energy used during the hibernating season, which is a prolonged state of food restriction [33]. In the grey mouse lemur, increased torpor expression is also associated with larger amounts of fat reserves [10]–[12], [21]; and as already demonstrated only winter-acclimated mouse lemurs exposed to a food-restricted period increase their torpor occurrence (Figure 1) [10]–[12], [21]. Therefore, mouse lemurs under winter phenotype may mainly use lipid mass, when exposed to a food restriction period. In fasting Svalbard ptarmigans, the sparing of body protein is more efficient in fat than lean birds, indicating that the initial body fat of animals plays a major role in determining the proportion of fat mass/fat-free mass oxidized during fasting [34]. This feature is all the more relevant under food deprivation since glycogen stores of the organism are partly replenished after each daily allotment. Along the journey, glycogen stores are rapidly depleted and lipid reserves are gradually mobilized for fuelling energy demands of the remaining daytime. Under food restriction, the low fat mass level of mouse lemurs in summer would be rapidly depleted, and therefore fat-free mass would be progressively used to meet energy needs. Interestingly, partially food-deprived Belding ground squirrels lost fat-free mass but not lipid mass during summer, indicating protein oxidation in lean animals [17].

Conversely, wintering mouse lemurs would mainly used during a moderate food shortage their large fat stores, sparing protein mass. Such conservation of muscle mass would be largely helpful for *M. murinus* during the wintering period. At the early beginning of the reproductive season, male mouse lemurs have to be immediately efficient and competitive to female access as soon as they emerge from wintering torpor [35]. Moreover, heavier males have greater opportunities to monopolize emerging females, and thus have higher reproductive rates than leaner animals [36]. Therefore, male mouse lemurs in winter may maintain sufficient level of fat-free mass to 1) keep muscle functionality and 2) preserve a high body mass level to be competitive in the beginning of the mating season.

### Energetic Consequences of Fat Sparing in Summer Acclimated Mouse Lemurs

In mouse lemurs in summer, which do not rely to increased torpor expression under moderate food-restricted conditions (Figure 1) [10]–[12], [21], the reduction in fat-free mass could fully explain the decrease in energy costs. Since the fat-free mass account for the major part of the energy consumption, this reduction constitutes the major mechanism, for mouse lemurs to reduce energy demands during a moderate food deprivation. Other species show similar responses. The Chilean mouse-opossum (*Thylamus elegans*) reduced maintenance costs mainly by reducing visceral mass, principally the digestive tract and liver [37]. These organs are probably the most expensive ones to maintain in term of energy and protein metabolism [38], [39]. Therefore, a reduction of fat-free mass, such as the size of energy-consuming organs, would contribute to maintain a stable energy balance during a moderate food scarcity in the grey mouse lemur.

Faced to a moderate food shortage, mouse lemurs under summer-like long-days would respond to food deprivation like all typical mammals, by decreasing protein synthesis and elevating protein catabolism, resulting in net nitrogen loss [40], [41]. In our study, although long-day mouse lemurs showed no changes in nitrogen balance and maintain a high nitrogen flux after 35 days of food deprivation, these animals might have had an early and acute negative nitrogen balance, during the early stage of the food deprivation, as suggested by the reduction in their body mass, during the first half of calorie deprivation. Therefore, the fact that summering mouse lemurs had attained nitrogen equilibrium, after a 35-days of food restriction was because they had already reached an adaptive low energy state. This lower active metabolic mass reduces energy demands and thus allows animals to downwardly adjust their energy balance to reflect the reduced body size while maintaining full ability to do physical activity. Doing so would allow for aggressive inter-male competitive behavior for female access [35], [42], [43], at the early beginning of the mating season, and so would contribute to a high rate of reproductive success, even during a moderate and early lowering of food supply.

In association with the reduction in metabolism triggered by the decrease in fat-free mass, water turnover was also reduced in mouse lemurs in summer facing a moderate food shortage. Our findings with the summering mouse lemurs are similar to those with the Arabian Oryx [44]. In Oryx, the rate at which water is processed is directly linked to its metabolic rate. Oryxes decrease their field metabolic rates by 50% and their water influx rates by 60% from spring to summer, concomitantly with a reduction in body mass [44]. Moreover, the decrease in water turnover described in *M. murinus* in summer under a moderate food shortage could fully be accounted by the reduced fat-free mass because the animals do not change their activity levels during food restriction [18].

### Protein Turnover Changes in Mouse Lemurs in Winter

Under *ad-libitum*, mouse lemurs in winter displayed a significantly reduced rate of protein turnover when compared to the summer state. Although some mechanisms underlying hibernation and torpor differ, similar phenomenon and potential explanations are found in hibernators. Protein synthesis and breakdown are both lower in wintering compared to summering black bears, indicating a lowered metabolism in animals in winter [45]. This seasonal difference in protein metabolism may be due to changes in the activity of molecular regulators that can differ in animals between seasons. The golden ground squirrel shows a seasonal variation in a potent regulator of an elongation factor that promotes protein translation in the liver. Summer squirrels lack this inhibitor, which down-regulates initiation of translation and thus protein expression, in wintering animals during hibernation [46].

Winter-like short-days mouse lemurs under a moderate food restriction were able to maintain their fat-free mass, which is likely to play a role in the torpor mechanism. During the cold and dry season in the Kirindy forest, mouse lemurs arouse from the low torpor state through a two-step process, consisting of an initial passive climb in Tb in relation to the ambient temperature followed by an active rise of Tb from 28–30°C to euthermic level [47], [48]. Therefore, the fat-free mass sparing occurring in the winter-acclimated animals, that faced a moderate food restriction, would likely contribute to the active reheating process of the body until normothermic values. Although an active rewarming involves the activation of the brown adipose tissue, resulting in non-shivering thermogenesis [13], [49], [50], other processes, for example a high level of active metabolic mass from the shivering mechanism, are likely to contribute.

In mouse lemurs in winter under a moderate food deprivation, the enhanced reduction in protein turnover would contribute to decreases in daily energy expenditure and water turnover. Maintenance of protein pools is the result of a balance between protein synthesis and degradation. In some tissues, protein synthesis and degradation account for as much as 20–40 and 4%, respectively, of oxygen consumption [19]. In a non-heterothermic rodent, inhibition of protein synthesis may reduce metabolic demands [51]. Hibernators also depress protein synthesis during their hibernating bout, slowing-down metabolic processes [52], [53]. Given that the torpor bouts reported in mouse lemurs are much shallower and shorter than those of a true hibernator, the 35% overall reduction of protein turnover would be only partially explained by the 5-fold increase in torpor expression in wintering animals. Concomitantly to a reduction of protein synthesis, hibernators would also decrease the rate of their protein catabolism, as suggested in the golden ground squirrel, by the accumulation of ubiquitylated proteins due to a decreased ability to degrade the tagged proteins [54]. Interestingly, the activity of protein degradation pathways during daily torpor was reduced in the Siberian hamster, through a down regulation of ubiquitylation-related transcripts [55]. This lowered activity under torpid state may be advantageous by reducing the overall energetic costs associated with protein degradation and would explain the decrease in protein catabolism in mouse lemurs in winter. Taken together, these data suggest that changes in protein turnover during torpor trigger an overall lowering of metabolism, through a reduction in enzyme activities, and consequently a conservation of energy and water.

Conversely to winter animals under moderate food shortage, mouse lemurs facing a severe food deprivation showed a significant chronic negative nitrogen balance and were unable to reduce their nitrogen flux, due to a dramatic rise in their protein catabolism. This increase led to a reduction in their fat-free mass and was similar to a food starvation response in term of protein use [56]. Nevertheless, due to their high initial level of fat mass, wintering mouse lemurs may also oxidize fatty acids to meet energy needs, under a severe food shortage, thereby limiting the rate of protein loss. This assumption is supported by the significant increase in normetanephrine, reflecting the overall activity of the sympathetic nervous system (SNS), found in this food-deprived animal group. During calorie restriction, activity of SNS is repressed in most tissues [57] except white adipose tissue, in which SNS activity is enhanced [58]. Therefore, as fat mass is progressively reduced, the proportion of energy derived from protein would gradually increase. This enhanced protein loss toward a critical mass, such as that probably reached by summering mouse lemurs under severe food shortage, would lead to an increased emergency signal and would trigger a high stress level in animals in winter, facing a severe 80% food deprivation. This later nutritional state of an overall stress, similar to fasting, is supported by the high level of metanephrine that constitute the hormonal signal of an emergency state.

### Conclusion

Under moderate food shortages, mouse lemurs use distinct efficient mechanisms of energy savings depending on the season. Nevertheless, when faced to a severe food scarcity, wintering mouse lemurs reached a critical state of a high stress level of and protein loss. This severe energy shortfall in the long term would affect the fitness and thus the survival of *M. murinus*, especially in the context of global changes in which the frequency and the intensity of such periods of food scarcity are predicted to increase.
